# Impact of Experimental Tonic Pain on Corrective Motor Responses to Mechanical Perturbations

**DOI:** 10.1155/2020/8864407

**Published:** 2020-07-31

**Authors:** Elodie Traverse, Clémentine Brun, Émilie Harnois, Catherine Mercier

**Affiliations:** ^1^Center for Interdisciplinary Research in Rehabilitation and Social Integration (CIRRIS), Quebec City, QC, Canada; ^2^Faculty of Medicine, Laval University, Québec, QC, Canada

## Abstract

Movement is altered by pain, but the underlying mechanisms remain unclear. Assessing corrective muscle responses following mechanical perturbations can help clarify these underlying mechanisms, as these responses involve spinal (short-latency response, 20-50 ms), transcortical (long-latency response, 50-100 ms), and cortical (early voluntary response, 100-150 ms) mechanisms. Pairing mechanical (proprioceptive) perturbations with different conditions of visual feedback can also offer insight into how pain impacts on sensorimotor integration. The general aim of this study was to examine the impact of experimental tonic pain on corrective muscle responses evoked by mechanical and/or visual perturbations in healthy adults. Two sessions (Pain (induced with capsaicin) and No pain) were performed using a robotic exoskeleton combined with a 2D virtual environment. Participants were instructed to maintain their index in a target despite the application of perturbations under four conditions of sensory feedback: (1) proprioceptive only, (2) visuoproprioceptive congruent, (3) visuoproprioceptive incongruent, and (4) visual only. Perturbations were induced in either flexion or extension, with an amplitude of 2 or 3 Nm. Surface electromyography was recorded from Biceps and Triceps muscles. Results demonstrated no significant effect of the type of sensory feedback on corrective muscle responses, no matter whether pain was present or not. When looking at the effect of pain on corrective responses across muscles, a significant interaction was found, but for the early voluntary responses only. These results suggest that the effect of cutaneous tonic pain on motor control arises mainly at the cortical (rather than spinal) level and that proprioception dominates vision for responses to perturbations, even in the presence of pain. The observation of a muscle-specific modulation using a cutaneous pain model highlights the fact that the impacts of pain on the motor system are not only driven by the need to unload structures from which the nociceptive signal is arising.

## 1. Introduction

Movement is altered in the presence of pain, but our theoretical models to explain these pain-induced motor adaptations still remain limited, as well as our understanding of the underlying neurophysiological mechanisms [1–4]. Adaptation to pain is known to involve changes at multiple levels of the motor system, and these changes can potentially be complementary, additive, or competitive [1]. Importantly, different types of pain may lead to different types of motor adaptations (e.g., tonic pain that is unrelated to movement vs. phasic pain that occurs in relation to a specific movement). In patients, tonic pain that may be aggravated by activity (but that does not occur in relation to specific movements) is observed mainly in patients with neuropathic pain, while phasic pain that is specifically associated with a given movement is more typically observed nociceptive musculoskeletal pain. While the effect of phasic musculoskeletal pain on movement is more intuitive and has been studied more extensively using experimental models such as hypertonic saline injection [1, 4], neuropathic pain that is unrelated to movement has been shown to be associated with motor alterations in clinical populations or clinical models (e.g., phantom limb pain or neuropathic pain in individuals with a spinal cord injury or a stroke) [2, 5]. Studying motor adaptation to this type of pain, using different experimental pain models such as capsaicin, is therefore also warranted.

A large number of studies using single-pulse transcranial magnetic stimulation have shown that pain, either phasic or tonic, can decrease corticomotor excitability [6–11], but this approach does not permit to distinguish between effects occurring at the cortical vs. at the spinal level. Only one study looked at motor responses evoked by stimulation applied both at the motor cortex and at the cervicomedullary junction, and the results obtained suggest reciprocal changes at the cortical (inhibition) and spinal (facilitation) level in the presence of pain [12]. However, results of studies focusing solely on the effect of pain at the spinal level are mixed, with studies reporting either an increase in H-reflex or stretch reflex amplitude, i.e., a spinal facilitation [12–14] or an absence of modulation [6, 15, 16]. Another limitation of the literature on the impact of pain on the motor system is that most studies are performed with the motor system at rest or involve only isometric contraction. This certainly limits our understanding of the interactions between the nociceptive and motor systems in a functionally relevant task, as well as of the potential interaction with information arising from other sensory modalities.

The use of sensory perturbations, such as a small mechanical perturbation applied to a static limb, is a powerful tool to alter the state of the sensorimotor system and observe how it responds to maintain control [17]. Detailed analysis of the muscle responses to such perturbations can provide an important clue of the level at which the motor system is affected by the context or sensory information [18–21]. The short-latency (SL) response, occurring from 20 to 50 ms, depends exclusively on spinal mechanisms and on proprioceptive signals. This response arises from the activation of I-afferent fibers and is target position-dependent and load-dependent [22, 23]. The long-latency response (LL; 50 to 100 ms) reflects both spinal and transcortical processes as it includes two distinct components. The first component arises from the activation of II-afferent fibers, and the second is the task-dependent component related to the integration of proprioceptive signal [20], especially by the cerebellum [23]. Finally, the early voluntary response (EV; 100 to 150 ms) results from a visuomotor control related to the integration of visual and proprioceptive afferents signals at the cortical level [21]. Importantly, measuring corrective responses to perturbations allows studying the whole sensorimotor system (rather than only the motor system), which is highly relevant given that pain is known to alter not only the state of the motor system but also somatosensory processing and proprioception [7, 24–26]. Sensory afferences are optimally integrated (according to the reliability/noise of each signal) [27, 28] in order to monitor and correct online body movements [29]. During feedback control, healthy individuals normally rely almost exclusively on proprioceptive feedback, even when visual information about limb motion is available [19]. However, if proprioceptive information becomes less reliable in the presence of pain, it could cause a greater reliance on other sources of information, such as visual information.

The general aim of this study was to examine the impact of experimental tonic pain on SL, LL, and EV responses of corrective muscle responses evoked by perturbations in healthy adults. The first objective was to evaluate whether the effect of the type of sensory feedback (visual and proprioceptive feedback) associated with the perturbation differed depending on the presence of pain. It was hypothesized that vision would be weighted relatively more heavily in the presence of pain. The corrective muscle responses to perturbations would be smaller, and the uncertainty about the estimated state decreases the response gain [30, 31]. This effect was expected to be stronger in conditions where a mismatch between vision and proprioception was present and for EV responses. The second objective was to determine whether the effect of pain on muscle responses evoked by a perturbation differs across flexor and extensor muscles (Biceps Brachii (BB) and Triceps Brachii (TB)). Based on previous evidence of a differential impact of experimental pain on flexors and extensors [32], it was hypothesized that pain would exert a greater influence on muscle responses of the flexor muscle than those of the extensor muscle.

## 2. Materials and Methods

### 2.1. Participants

16 right-handed healthy participants (7 males, 9 females, age (mean ± standard deviation (SD)) = 26 ± 6.8 years) were recruited from the Laval University community in the Quebec City area. Exclusion criteria were (1) history of neurological or psychiatric disease, (2) presence of chronic or acute pain, (3) history of musculoskeletal injury to the right upper limb (UL), and (4) body mass index above 30 (this criteria being related to the difficulty to fit individuals with a higher body mass index in the experimental setup). All participants provided written informed consent in accordance with the Declaration of Helsinki (1964), and the study was approved by the Centre intégré universitaire de santé et de services sociaux de la Capitale-Nationale Human Ethics Committee (Project #2019-1736).

### 2.2. Study Design and Experimental Conditions

**Figure 1:**
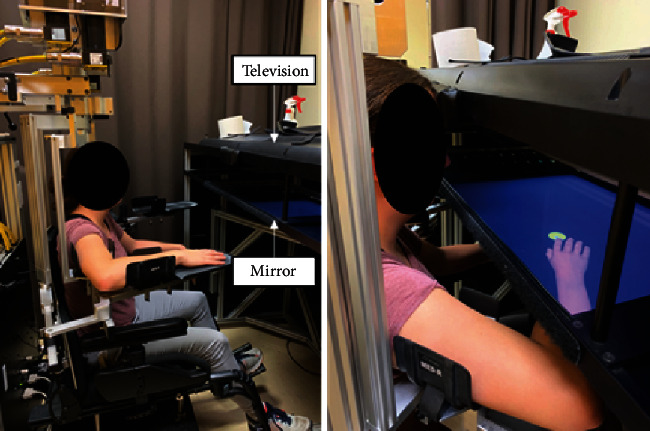
Participants were seated in a KINARM robotic exoskeleton allowing shoulder and elbow joint movements in the horizontal plane. The 2D virtual environment (television that is projected on a semitransparent mirror) associated with the exoskeleton allowed replacing the participants' right UL by a virtual UL. The initial joint configuration was 45° for the shoulder and 90° for the elbow.

The experimental paradigm employed in this study is based on a paradigm developed by Crevecoeur et al. [19]. Experiments were performed using a KINARM robotic exoskeleton Lab™ (KINARM, Kingston, Ontario), allowing shoulder and elbow joint movements in the horizontal plane (Figure 1). This system allowed the application of both mechanical perturbations and visual perturbations, using the 2D virtual environment associated with the exoskeleton to replace the actual upper limb (UL; always obstructed from view) by a virtual UL. The participants were instructed to maintain their index in a target despite the application of mechanical perturbations.

**Figure 2:**
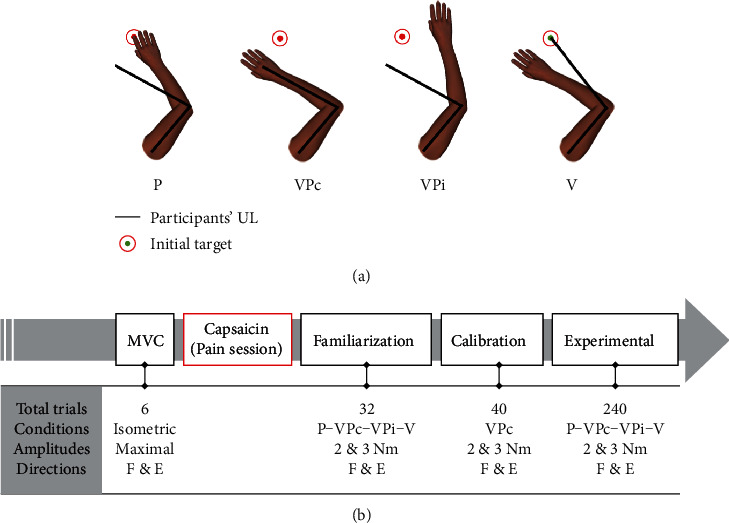
(a) Experimental sensory conditions. The illustration shows the discrepancy between the position of the virtual upper limb (UL) and the participants' UL (hidden from view, represented by black bars) in all experimental conditions except VPc. Note that the virtual UL and the participants' UL were always aligned on the initial target prior to the perturbation. (b) Study design. Study design comprised five main steps. Maximal voluntary contraction (MVC) and familiarization and calibration phases were needed for methodological purposes only. Experimental trials were performed in 5 blocks of 48 trials (a total of 240 trials), with a ~2-minute rest between each block. P = proprioceptive; VPc = visuoproprioceptive congruent; VPi = visuoproprioceptive incongruent; V = visual; F = flexion; E = extension.

Participants performed the same experimental paradigm over two sessions (randomized order; separated by 10.5 ± 6.5 days; ~2 hours/session), with (Pain session) or without (No pain session) the induction of experimental pain with a thin layer (~1 mm) of 1% capsaicin cream forming a 1 cm wide ring applied around the upper arm (just above the elbow crease) of the right UL. Capsaicin creates penetrating and irradiating burning sensations, reproducing neuropathic pain [33]. This specific pain model has been shown to induce pain level that is stable over time, irradiates through the whole elbow region, and can induce motor learning and sensorimotor integration deficits [34, 35]. In each session, the participant was exposed to four sensory conditions (Figure 2(a)):

1. Proprioceptive condition (P) involved only a mechanical perturbation, with the virtual UL staying still
2. Visuoproprioceptive congruent condition (VPc) involved a mechanical perturbation + a displacement of virtual UL similar to that of the actual UL
3. Visuoproprioceptive incongruent condition (VPi) involved a mechanical perturbation + a displacement of virtual UL similar to that of the actual UL, but in the opposite direction
4. Visual condition (V) involved a displacement of virtual UL similar to that induced by mechanical perturbations, but without any mechanical perturbation (control condition, no motor correction required)

Perturbations were applied in two directions (elbow flexion or extension) and with two different amplitudes (2 or 3 Nm) to increase unpredictability (and therefore the difficulty) of the experimental task. Overall, this resulted in 16 experimental conditions: [sensory conditions (“proprioceptive (P)” or “visuoproprioceptive congruent (VPc)” or “visuoproprioceptive incongruent (VPi)” or “visual (V)”)] × [amplitude (“2 Nm” or “3 Nm”)] × [direction (“flexion” or “extension”)].

At the beginning of each session, participants were installed in the KINARM and a calibration procedure was performed for their right UL. The experimental session then comprised five steps (Figure 2(b)):

1. Maximal voluntary isometric contraction (MVC) was assessed in elbow flexion and extension (3 trials/direction; >1 min rest between trials; order randomized), with the right UL in the horizontal plane (position similar to that of the experimental task)
2. Experimental tonic pain was induced, if applicable (Pain session only). Participants verbally rated pain intensity every 5 minutes for 20 minutes using a numeric pain rating scale (NRPS) ranging from 0 (No pain) to 10 (worst pain imaginable) (both sessions). After 20 minutes, participants started the next step, and pain intensity ratings were required after subsequent steps
3. Familiarization trials with the experimental task were realized (a total of 32 trials: 4 sensory conditions × 2 amplitudes × 2 directions × 2 trials)
4. Calibration trials in the VPc condition only were performed (a total of 40 trials: 2 amplitudes × 2 directions × 10 trials) to measure the hand paths following each mechanical perturbation. This was used to individually adjust the trajectory of the virtual UL displayed during the V condition
5. Experimental trials in all conditions were performed (a total of 240 trials: 4 sensory conditions × 2 amplitudes × 2 directions × 15 trials; pseudorandom counterbalanced order). Trials were divided into 5 blocks of 48 trials, with ~2 minutes of rest between each block. Pain intensity was reported immediately after each block of trials

### 2.3. Experimental Task

At the beginning of each trial, participants were instructed to place their index in the initial target (radius of 0.6 cm) projected on the virtual reality display. The position of this target corresponded to a joint configuration of 45° of horizontal abduction at the shoulder and 90° of flexion at the elbow (Figure 1). At this stage of the trial, the virtual UL was coupled with the participants' UL movement. Initially, the initial target was red and once the index was correctly positioned, it was turning green and a mechanical and/or visual perturbation was applied after a random delay ranging from 2 to 4 seconds. Participants were instructed to perform a motor correction in order to come back as quickly as possible in the initial target (the radius of this target increased from 0.6 to 2 cm). Importantly, a motor correction was required only when a mechanical perturbation was applied (P, VPc, and VPi conditions). In these conditions, the trial was considered valid only if a motor correction was performed within a delay of 800 ms following the perturbation. In the V condition, no motor correction was required. See Figure S1 in the Supplementary Material for videos providing examples of each sensory condition.

Mechanical perturbations consisted in equal step torque (20 ms buildup) applied with varying amplitudes (±2 Nm or ±3 Nm) on the shoulder and elbow, generating a pure elbow motion [19, 36]. For the visual perturbations, the virtual UL followed a trajectory fitted to participants' individual hand paths following 2 and 3 Nm mechanical perturbations in the two directions (flexion and extension) recorded during 40 calibration trials (Figures 2(a) and 2(b), VPc condition × 2 amplitudes × 2 directions × 10 trials). The fit was composed of four Gaussian functions fitted to *x*- and *y*-coordinates of the hand motion using MATLAB software (version R2013a). Then, the coordinates were input on the Dexterit-E software (IBM 3.7.2) for the virtual UL to reproduce the real UL motion.

### 2.4. Electromyographic Recordings

Electromyographic (EMG) activity was recorded from two muscles of the right UL (Biceps Brachii; long head of Triceps Brachii) using silver-chloride surface electrodes (220 mm diameters, Ag-AgCl, Kendall™ H69P, Covidien). Bipolar surface electrodes (interelectrode center-to-center distance of 2 cm) were placed on the midmuscle belly with the right arm placed in a horizontal plane. The reference electrode was placed on the external condyle of the right elbow. Before electrode placements, the skin was shaved and cleaned with alcohol to obtain low impedance (<5 kHz). The EMG signal was amplified (gain 1000), band-pass filtered (10 to 500 Hz), and digitized at a sampling rate of 1 kHz using the KINARM data acquisition card (National Instruments PCI-6229 DAQ card, Austin, TX, USA).

### 2.5. UL Kinematic Recordings

UL kinematic data (elbow angular acceleration, velocity, and position) were obtained from the KINARM motor encoders and sampled at 1 kHz.

## 3. Data Analyses

### 3.1. Data Preprocessing

For MVC, the root mean square (RMS) of BB and TB EMG (in flexion and extension, respectively) was calculated using a 50 ms moving window. The peak value was determined for each trial and then averaged across trials.

For experimental trials, muscle responses following extension perturbations for BB muscle and following flexion perturbations for TB muscle were considered. The RMS EMG of each muscle, normalized against MVC, was calculated for three time windows following the perturbation: (1) 20 to 50 ms for SL response, (2) 50 to 100 ms for LL response, and (3) 100 to 150 ms for EV response. In total, 4.4% of all data were rejected from analyses because experimental trials were invalid (no motor correction performed within 800 ms) or because of EMG artifacts. Preprocessing has been performed offline using MATLAB software (R2015b).

The changes in SL, LL, and EV normalized corrective motor responses (i.e., difference between Pain and No pain sessions) were also calculated.

### 3.2. Statistical Analyses

Data distributions were tested using the Kolmogorov-Smirnov test with the Lilliefors correction.

For pain intensity (normal distribution), the paired *T*-test was used to compare the first and the last experimental blocks in order to assess the stability of pain over the experiment.

For MVC (normal distribution), paired *T*-tests were used to compare the No pain and Pain sessions. For muscle responses, data were not normally distributed, and therefore, nonparametric analysis methods were employed. It was first verified whether muscle responses in each time window (SL, LL, and EV) involving a mechanical perturbation (P, VPc, and VPi) differed from the control condition without mechanical perturbation (V) using Wilcoxon tests. The V condition was not used for further analyses. Analyses related to objective 1 and objective 2 were performed using nonparametric analyses of variance (nonparametric for longitudinal data (nparLD)). NparLD is a robust method for factorial designs with small and inequivalent samples and does not require normality of distributions and homoscedasticity [37]. Importantly, only two within-factors can be tested in any given analysis [37]. For objective 1, by focusing on whether the effect of the type of sensory feedback (visual and proprioceptive feedback) associated with the perturbation differed depending on the presence of pain, different analyses assessing the effect of the sensory conditions (P, VPc, and VPi) and sessions (No pain and Pain) on the muscle responses were therefore performed for each muscle (BB and TB), each latency (SL, LL, and EV), and each amplitude (2 and 3 Nm). For objective 2, by focusing on whether the effect of pain varies across muscles, different analyses assessing the effect of the muscles (BB and TB) and sessions (No pain and Pain) on the muscle responses were performed for each latency (SL, LL, and EV) and each amplitude (2 and 3 Nm).

A posteriori, we conducted Spearman's correlations to evaluate the relationship between the intensity of pain and the changes in SL, LL, and EV muscle responses to perturbation (i.e., difference between Pain and No pain sessions). When a significant association was found, supplementary nonparametric analyses similar to those performed for objective 2 were performed on subgroups with low pain vs. high pain (created using a median split on pain intensity ratings, with median pain intensity being 3.7/10).

For all analyses, the level of significance for *p* values was set to 0.05. No correction was applied for multiple tests and post hoc analyses. Statistical tests were realized with the IBM SPSS statistic software (v26), except for nparLD tests that were performed with the R software (version 3.5.2) using the nparLD package [37].

## 4. Results

### 4.1. Pain Intensity

**Figure 3:**
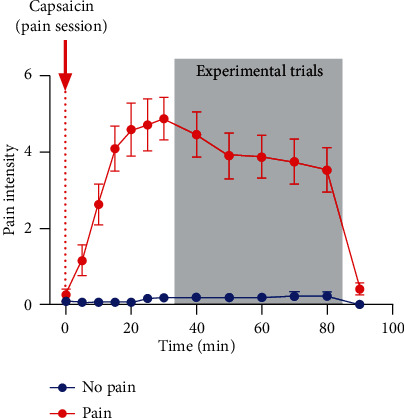
Average pain intensity over time ranging from 0 (No pain) to 10 (the worst pain imaginable) during the Pain (red) and No pain (blue) sessions. For the Pain session, the capsaicin was applied at time 0 (red arrow). The shaded area represents the time during which experimental trials were performed. Error bars represent the Standard Error of Mean (SEM).

Figure 3 shows the evolution of pain intensity over time for the No pain session (mean of 0.2/10 ± 0.4) and for the Pain session (mean of 3.9/10 ± 2.3). The paired *T*-test demonstrated a significant decrease in pain intensity over time during experimental trials of the Pain session (*t*_15_ = 2.88, *p* = 0.01). Nevertheless, a substantial level of pain was still reported at the end of the last experimental block (mean ± SD: 3.5 ± 2.3). The mean pain intensity for each subgroup created for a posteriori analyses was 1.9/10 ± 0.9 in the low pain group and 5.9/10 ± 1.2 in the high pain group.

### 4.2. Maximal Voluntary Contraction

As MVC from each session was used for normalization purposes, it was first verified that there was no systematic difference between No pain and Pain sessions. No difference was observed for either BB (*p* = 0.50) or TB (*p* = 0.31) muscle.

### 4.3. Muscle Responses to Perturbation

**Figure 4:**
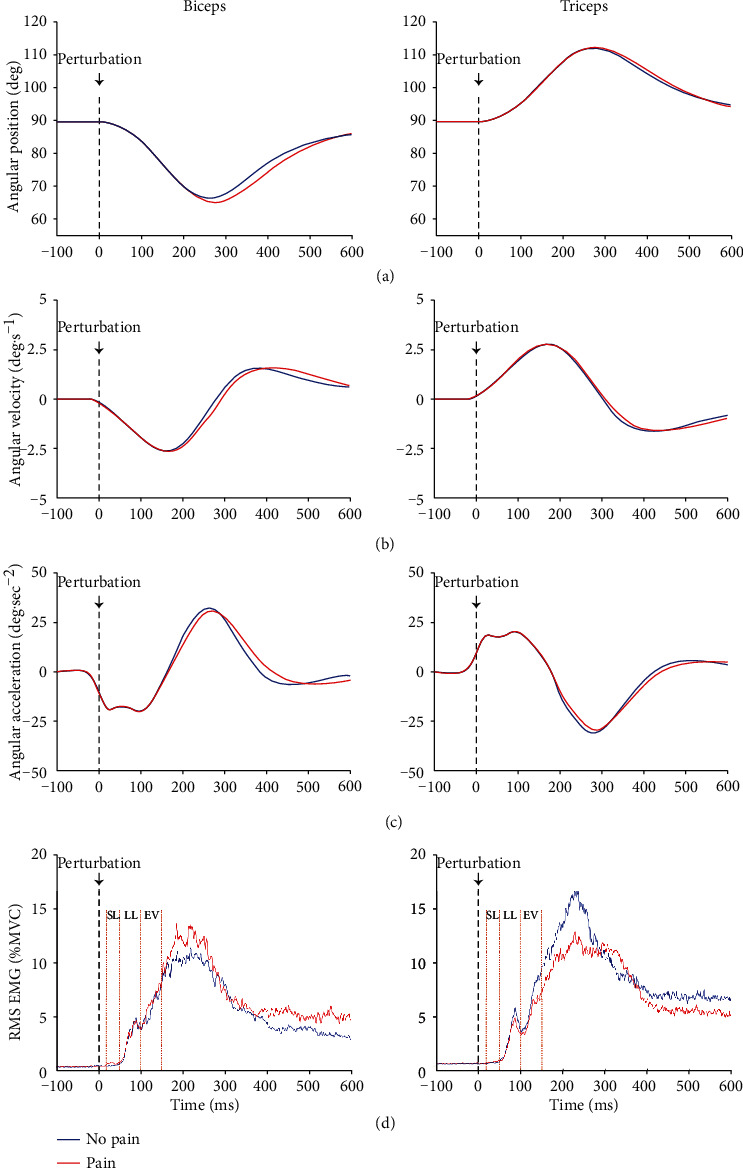
Average traces of elbow angular position (a), velocity (b), acceleration (c), and muscle responses (d) for Biceps (left) and Triceps (right) during experimental trials (average of P, VPc, and VPi sensory conditions). The short-latency (SL) response, occurring from 20 to 50 ms, provides information on spinal mechanisms and depends exclusively on proprioceptive signals. The long-latency response (LL; 50 to 100 ms) reflects both spinal and transcortical processes related to the integration of proprioceptive signals, especially by the cerebellum. The early voluntary response (EV; 100 to 150 ms) results from a visuomotor control related to the integration of visual and proprioceptive afferent signals at the cortical level. The period between 150 ms and 600 ms, during which participants were actively maintaining their index on the initial target against the perturbation, was not included in the analyses as this represents voluntary muscle activity. MVC = maximal voluntary contraction.

Figure 4 depicts average traces of normalized RMS EMG (across all participants and experimental sensory conditions involving mechanical perturbations (P, VPc, and VPi)). Results (graphs and statistics) are presented only for the 3 Nm amplitude as stronger mechanical perturbations elicited larger muscle responses. Nevertheless, results for the 2 Nm amplitude show the same pattern.

Table 1 shows the results for the SL, LL, and EV responses in each muscle, each sensory condition, and each session. Muscle responses during P, VPc, and VPi conditions were compared to those during the V condition to ensure that the muscle responses statistically exceeded the background EMG (i.e., in the absence of mechanical perturbation). This was the case even for the SL muscle response (all *p* < 0.05), although clear SL responses are difficult to distinguish in Figure 3(d).

For objective 1, nparLD analyses on BB and TB muscle responses revealed no significant main effect of condition and session and no significant interaction between conditions (P, VPc, and VPi) and sessions (No pain and Pain) for either SL, LL, or EV responses (all *p* > 0.08).

**Figure 5:**
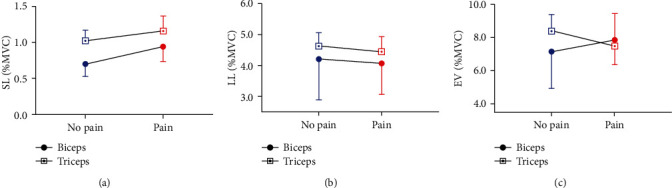
Mean ± Standard Error of Mean (SEM) for RMS EMG in SL (a), LL (b), and EV (c) responses expressed as percentage of maximal voluntary contraction (%MVC) during Pain (red) and No pain (blue) sessions for Biceps (circle) and Triceps (square) muscles.

For objective 2, by focusing on whether the effect of pain varies across muscles, different analyses assessing the effect of the muscles (BB and TB) and sessions (No pain and Pain) on the muscle responses were performed for each latency (SL, LL, and EV). As no significant differences were observed between sensory conditions in objective 1, muscle responses at each latency were averaged across P, VPc, and VPi sensory conditions.  Figure 5 depicts SL (Figure 5(a)), LL (Figure 5(b)), and EV (Figure 5(c)) responses for each muscle during each session. Results show a significant main effect of muscle (responses being larger in TB compared to BB) for SL (*p* = 0.017) and LL (*p* = 0.001) but not for EV (*p* = 0.073) responses. The effect of the session was not significant for SL, LL, and EV responses (for all, *p* > 0.2). Finally, a significant interaction was found between muscle and session for the EV response (*p* = 0.005) but not for the SL (*p* = 0.31) and the LL (*p* = 0.29) responses. However, post hoc analyses for EV responses were not significant (all *p* > 0.1).

**Figure 6:**
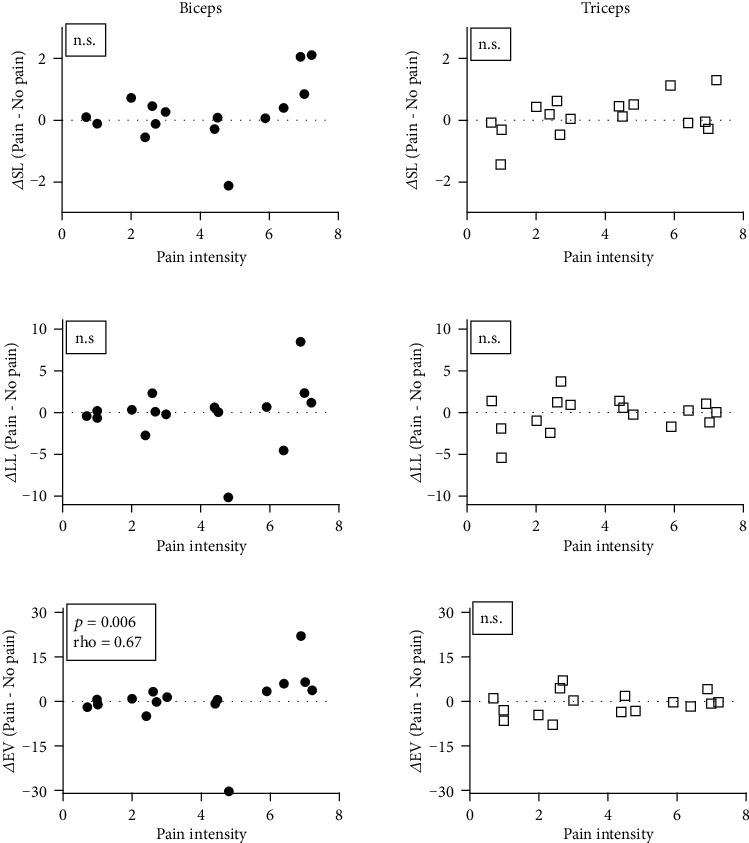
Association between pain intensity and the pain-induced changes in SL, LL, and EV responses (*∆*) for biceps and triceps muscles. These changes were calculated as the difference between normalized corrective responses during the Pain session and during the No pain session for each participant. Positive values showed an increase in muscle responses during the Pain session whereas negative values showed a decrease in muscle responses during the Pain session compared to the No pain session. Participants verbally rated pain intensity using a numeric pain rating scale ranging from 0 (no pain) to 10 (worst pain imaginable).

**Figure 7:**
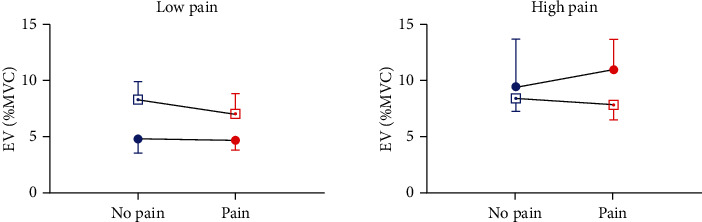
Mean ± Standard Error of Mean (SEM) for RMS EMG in EV responses expressed as percentage of maximal voluntary contraction (%MVC) during Pain (red) and No pain (blue) sessions for Biceps (circle) and Triceps (square) muscles.

A posteriori analyses showed no significant correlation between the intensity of pain and the changes in SL and LL responses between Pain and No pain sessions (all *p* > 0.09). However, a positive significant relationship was found for EV responses from the BB muscle (*p* = 0.006, rho = 0.67), but not from the TB muscle (Figure 6). NparLD analyses conducted for objective 2 were then performed again for EV responses in the BB muscle only, separately for subjects exhibiting high pain and low pain (median split). Results from the high pain group reproduced those obtained with the complete group, and no significant main effect of muscle and session (*p* > 0.42) was found, but results showed a significant interaction (*p* = 0.001) (Figure 7). Post hoc analyses were not significant (all *p* > 0.2). Results for the low pain group did not show significant main effects or interaction between muscle and session (all *p* > 0.09).

## 5. Discussion

The general aim of this study was to examine the impact of experimental tonic pain on SL, LL, and EV responses of the corrective muscle responses evoked by perturbations in healthy adults. The first objective was to evaluate whether the effect of the type of sensory feedback (visual and proprioceptive feedback) associated with the perturbation differed depending on the presence of pain. Results demonstrate that corrective muscle responses are not influenced by the type of sensory feedback, no matter whether pain is present or not. The second objective was to determine whether the effect of pain on muscle responses evoked by a perturbation differs across flexor and extensor muscles. Results show that corrective responses in flexor and extensor muscles exhibit a differential modulation in the presence of pain, and particularly of higher pain intensity, but only for EV responses.

For objective 1, it was hypothesized that the EV responses to perturbations would be smaller, as proprioceptive feedback would be less reliable. This effect was expected to be stronger in conditions where a mismatch between vision and proprioception was present, for EV responses specifically, but our hypothesis was not supported by the results. The fact that no main effect of the conditions (P, VPc, and VPi) was observed confirms previous findings showing that EV responses are mainly based on proprioception [19, 38] and extend them by demonstrating that this still holds when there is a clear mismatch between visual and proprioceptive information (i.e., movement occurring in opposite directions). This contrasts with studies on self-initiated voluntary movements showing that discordance between visual feedback and voluntary movement induces motor perturbations [34, 39, 40]. This discrepancy between the two types of task is probably explained by the fact that corrective muscle responses need to be performed very fast, while the motor tasks using self-initiated voluntary movements are generally slow. An important factor to consider for optimal sensory integration besides signal noise is the fact that there are delays in transmitting sensory information. Visual feedback is relatively accurate but slow, whereas proprioceptive feedback is less accurate but fast [19]. This creates a trade-off between speed and accuracy, and the respective contribution of visual and proprioceptive information can be flexibly adjusted according to the situation [38, 41, 42]. The lack of interaction between the presence of pain and the condition of feedback suggests that the need for a rapid response supersedes the potential impact of pain on the processing of proprioceptive information, despite some evidence that pain makes people more sensitive to visuomotor conflicts [34, 43–47].

For objective 2, it was hypothesized that pain would exert a greater influence on responses of the flexor muscle than those of the extensor muscle. No differential effect of pain across muscles was observed for SL and LL responses, and in fact, no influence of pain at all was observed for these responses, suggesting that experimental tonic pain does not affect the excitability at the spinal level. As mentioned earlier, previous results using stimulation at the cervicomedullary junction, H-reflex, or stretch reflex to study the effect of pain on spinal excitability are conflicting [6, 12, 13, 15, 16]. Discrepancies might be explained by the different experimental pain models used across these studies. For instance, studies that reported spinal facilitation [12, 13] used intramuscular hypertonic saline injection. This experimental pain model activates muscle nociceptive receptors (A*δ* and C afferences) [33] and has been shown to impact Ia and II neuromuscular spindle afferences [48] involved in short- and long-latency muscle responses [20]. However, topical application of capsaicin cream activates cutaneous nociceptive receptors, mainly C and A*β* afferences, but not A*δ* [33]. Therefore, the discrepancies across studies might be explained by the fact that the activation of muscle nociceptive afferences, but not of nociceptive afferents from skin, modulates spinal excitability. Indeed, the only other study that assessed the effect of capsaicin on the spinal excitability (H-reflex amplitude) reported no modulation [6], similar to the results of the present study. The modality appears to matter more that the pain intensity, as average pain ratings are comparable across studies using topical capsaicin cream [49–53] and intramuscular hypertonic saline injection [12, 13, 16]. Importantly, several previous studies using experimental cutaneous pain demonstrated modulations of corticospinal excitability [6, 49–52] and alterations of motor performance [54] and motor learning [55, 56]. Therefore, the present results should not be interpreted as indicating that cutaneous pain does not interact with the motor system, but rather that in the case of cutaneous pain, these interactions appear to occur at the cortical level rather than at the spinal level.

For EV responses, a significant interaction between the presence of pain and the muscle tested was observed. We did not observe a greater effect of pain on the flexor muscle but rather an opposite pattern of modulation caused by pain (toward facilitation for biceps and toward inhibition for triceps). Importantly, the post hoc analyses were not significant, which suggests that the influence of pain was modest. However, analyses performed a posteriori demonstrated that the pain-induced changes in muscle responses depended on the intensity of this pain, especially for flexor muscle. Indeed, the interaction observed at the group level was reproduced in the high pain subgroup, but not in the low pain subgroup. A recent study demonstrated a greater decrease in corticospinal excitability for laser stimuli of higher intensity associated with higher pain level [57], consistent with the results of this study while using another experimental pain model.

Nevertheless, the presence of a clear interaction for the EV response in the absence of effect for earlier responses suggests that in this type of task, interaction between pain and motor control occurs at the cortical level only and in a muscle-dependent manner. In addition to the large brain network which is classically associated with pain processing (including the primary and secondary somatosensory, insular, and anterior cingulate and prefrontal cortices and thalamus), some functional neuroimaging studies have reported hemodynamic changes in brain regions related to motor function during pain, including the primary motor cortex [58–60]. As mentioned in Introduction, many transcranial magnetic stimulation studies also showed a pain-induced modulation of corticospinal excitability [6, 49–51], with some studies showing alterations in mechanisms which are known to be intracortical, such as short-interval intracortical inhibition [61–63] and interhemispheric inhibition [64]. Only a few transcranial magnetic stimulation studies examined the effect of pain specifically on circuits involved in sensorimotor integration, which is relevant to EV responses. While no alterations were observed in short- and long-latency afferent inhibition during exposure to muscle pain or heat cutaneous pain [11, 65], these two mechanisms were found to be altered once muscle pain has resolved [65]. Neurophysiological studies have typically focused on a single muscle due to the need to optimize stimulation parameters for a given muscle. However, a recent study showed muscle-dependent modulation of corticospinal excitability during the motor preparation phase of a movement that is expected to cause pain. While there is ample evidence that the presence of intramuscular pain induces redistributions of muscle activity across agonist, synergist, and antagonist muscles [3, 66], the observation of a muscle-dependent modulation prior to the occurrence of the nociceptive stimuli [32] or with a cutaneous pain model (in the present study) suggests that the interaction between the nociceptive and motor systems is not simply driven by the need to unload structures from which the nociceptive signal is arising.

## 6. Conclusions

Overall, results of this study show that the impact of experimental cutaneous pain on corrective muscle responses evoked by perturbations in healthy adults is limited to a muscle-specific modulation of EV responses. The lack of effect on earlier muscle response of corrective responses suggests that spinal mechanisms are not affected by experimental cutaneous pain, which might differ from other pain models activating muscular nociceptive afferences. Despite the fact that pain has been shown to interfere with proprioception and to make people more sensitive to sensorimotor conflicts, no influence of the type of sensory feedback on corrective muscle responses was found, no matter whether pain is present or not. This might be explained by the need for a rapid response in the presence of a perturbation and underline the need to study a variety of motor tasks to develop a comprehensive understanding of interactions between the nociceptive and motor systems. An important finding is the presence of muscle-specific modulation using a cutaneous pain model, which should raise awareness about the fact that the impacts of pain on the motor system are not only driven by the need to unload structures from which the nociceptive signal is arising.

## Figures and Tables

**Table 1a:** (a) Biceps

Session	Response	Condition				
		Experimental				Control
		P	VPc	VPi	Mean	V
No pain	SL	0.69 ± 0.68	0.69 ± 0.66	0.72 ± 0.81	0.70 ± 0.71^∗∗^	0.56 ± 0.66
	LL	4.01 ± 5.54	4.06 ± 4.44	4.52 ± 5.93	4.20 ± 5.27^∗∗∗^	0.56 ± 0.67
	EV	7.13 ± 8.83	6.11 ± 5.13	8.15 ± 13.1	7.13 ± 8.88^∗∗∗^	0.57 ± 0.68
Pain	SL	0.96 ± 0.89	0.94 ± 0.83	0.91 ± 0.75	0.94 ± 0.82^∗∗^	0.62 ± 0.54
	LL	4.07 ± 4.07	4.05 ± 3.72	4.05 ± 4.26	4.06 ± 3.99^∗∗∗^	0.74 ± 0.91
	EV	7.72 ± 6.33	7.56 ± 5.79	8.19 ± 7.08	7.83 ± 6.36^∗∗∗^	0.66 ± 0.62

**Table 1b:** (b) Triceps

Session	Response	Condition				
		Experimental				Control
		P	VPc	VPi	Mean	V
No pain	SL	1.02 ± 0.61	1.04 ± 0.62	1.00 ± 0.55	1.02 ± 0.59^∗∗∗^	0.87 ± 0.55
	LL	4.61 ± 1.75	4.65 ± 1.88	4.59 ± 1.86	4.62 ± 1.73^∗∗∗^	0.88 ± 0.55
	EV	8.38 ± 3.78	8.34 ± 3.93	8.35 ± 4.11	8.36 ± 3.89^∗∗∗^	0.88 ± 0.53
Pain	SL	1.18 ± 0.86	1.14 ± 0.80	1.15 ± 0.80	1.16 ± 0.81^∗^	1.01 ± 0.75
	LL	4.43 ± 2.02	4.37 ± 1.75	4.49 ± 2.30	4.43 ± 1.98^∗∗∗^	0.99 ± 0.71
	EV	7.58 ± 4.63	7.49 ± 4.55	7.28 ± 4.30	7.45 ± 4.40^∗∗∗^	1.02 ± 0.72

## Data Availability

The EMG data used to support the findings of this study are available from the corresponding author upon request.

## Supplementary Materials

Supplementary Materials Additional supporting information may be found in the online version of this article. Figure S1: video of experimental sensory conditions. The video shows the discrepancy between the position of the virtual UL and the participants' UL (hidden from view, represented by blue bars) in all experimental conditions (P, VPc, VPi, and V). Note that the condition illustrated was performed in flexion or extension direction, following visual or mechanical perturbations at 3 Nm amplitude.
